# Have policy responses in Nigeria resulted in improvements in infant and young child feeding practices in Nigeria?

**DOI:** 10.1186/s13006-017-0101-5

**Published:** 2017-02-08

**Authors:** Felix A. Ogbo, Andrew Page, John Idoko, Fernanda Claudio, Kingsley E. Agho

**Affiliations:** 1 Centre for Health Research, School of Medicine, Western Sydney University, Campbelltown Campus, Locked Bag 1797, Penrith, NSW 2571 Australia; 2 Department of Medicine, Faculty of Medical Sciences, University of Jos, P.M.B 2084, Jos Abuja, Plateau State Nigeria; 3 School of Medicine, The University of Queensland, Brisbane, QLD 4072 Australia; 4 School of Science and Health, Western Sydney University, Campbelltown Campus, Locked Bag 1797, Penrith, NSW 2571 Australia

**Keywords:** Breastfeeding, Complementary feeding, Infant and young child, Nigeria, Policy

## Abstract

**Background:**

Nigeria initiated a range of programs and policies (from 1992 to 2005) to improve infant and young child feeding (IYCF) practices. However, the prevalence of children fed in accordance with IYCF recommendations in Nigeria remains low. This paper presents time trends in IYCF practices in Nigeria for the period (1999–2013), and considers trends in the context of key national policy responses and initiatives.

**Methods:**

Prevalence and percentage change (including 95% confidence intervals) of IYCF indicators were investigated over the period 1999–2013 based on a total of 88,152 maternal responses from the Nigeria Demographic and Health Surveys, (*n* = 8,199 in 1999; *n* = 7,620 in 2003; *n* = 33,385 in 2008 and *n* = 38,948 in 2013).

**Results:**

Early or timely initiation of breastfeeding decreased significantly by 4.3% (95% Confidence Interval [CI]: −8.1, −0.5; *p* = 0.0280 for the period (1999–2013); while exclusive breastfeeding remained unchanged 1.6% (95% CI: −2.7, 5.9; *p* = 0.478). From 2003 to 2013, minimum meal frequency increased significantly by 13.8% (95% CI: 9.9, 17.8; *p* < 0.001), but minimum dietary diversity and minimum acceptable decreased significantly by 9.7% (95% CI: −9.2, −6.3; *p* < 0.001) and 3.5% (95% CI: −5.7, −1.3; *p* = 0.002), respectively. Predominant breastfeeding increased significantly by 13.1% (*p* < 0.001), and children ever breastfed declined by 16.4% (*p* < 0.001) over time.

**Conclusion:**

Despite considerable improvements in national legislation, health system responses and community level development, IYCF practices in Nigeria are still below expected levels. Strengthening community and facility based participation, and broader stand-alone/integrated IYCF policy implementations are needed to improve the current feeding practices of Nigerian mothers.

**Electronic supplementary material:**

The online version of this article (doi:10.1186/s13006-017-0101-5) contains supplementary material, which is available to authorized users.

## Background

Appropriate and safe infant and young child feeding (IYCF) practices have been shown to improve child health and development, including changing nutritional needs [1, 2]. Based on the benefits of optimal feeding practices in early childhood, the World Health Organisation and United Nation Children’s Fund (WHO/UNICEF) recommend the initiation of breastfeeding for all newborns within the first hour of life, exclusive breastfeeding (EBF) for the first six months of life, and continued breastfeeding for two years and beyond with nutritionally appropriate and safe complementary foods introduced around the sixth month [3, 4].

Following the WHO/UNICEF recommendations on improving infant and young child feeding practices, Nigeria initiated several programs and policies responses to promote and support infant and young child feeding practices. These included the Baby Friendly Hospital Initiative (BFHI) in 1992 [5], the National Breastfeeding Policy in 1998 [6], the National Policy on Food and Nutrition in 2001 and the National Policy on Infant and Young Child Feeding in 2005 [7]. Some improvements were observed in early or timely initiation of breastfeeding following the introduction of the BFHI, from 31.4% in 1990 to 39.2% in 2008 [8].

In comparison to other developing countries such as Ethiopia, India and Indonesia where the national policy, strategy, and plans of action and health system framework to improve IYCF practices are poor, Nigeria has an established a national legislative and health system framework to promote and support infant and young child feeding practices [9]. Despite these initiatives, malnutrition, and early childhood feeding related diseases and mortality still remain problems of public health importance in Nigeria [10–12]. For example, there are approximately 2,300 deaths per day among children under five years in Nigeria attributable mainly to preventable diseases, and suboptimal infant feeding practices play a major role [13]. Furthermore, there has been a drop in the proportion of children under 24 months of age who were fed in accordance with IYCF (breastfeeding and complementary feeding) guidelines in Nigeria, from 30% in 2009 [14] to 10% in 2014 [12].

Country specific data are needed to advocate for and to guide more effective and sustainable national health policies and initiatives, to monitor interventions that aim to improve infant and young child feeding practices, and also to evaluate the impact of previous initiatives. Thus, this paper presents time trends in nationally representative infant and young child feeding (IYCF) practices in Nigeria for the period 1999–2013, spanning the development and implementation of a range of key national policy responses and initiatives. The paper also aims to demonstrate the need for context specific maternal and child health policy reform and funding in Nigeria to encourage appropriate feeding practices in Nigerian mothers. Additionally, evidence from this paper may be used to advocate for specific interventions needed in achieving the health-related Sustainable Development Goals (SDG) in Nigeria.

## Method

### Data sources

The present study used the Nigeria Demographic and Health Surveys (NDHS), collected by the National Population Commission (NPC) and Inner City Fund (ICF) International [12, 14–16]. Trends in IYCF practices in Nigeria were assessed using the World Health Organisation (WHO) definitions for IYCF indicators, categorized as core or optional indicators, for assessing infant and young child feeding practices in a population as described below. These indicators were assessed based on a 24 h recall, consistent with the WHO definitions [4]. A total of 88,152 (*n* = 8,199 in 1999, *n* = 7,620 in 2003, *n* = 33,385 in 2008 and *n* = 38,948 in 2013) maternal responses from the NDHS data were examined, with response rates ranging from 92–98% [12, 15]. The increase in sample size in 2008 and 2013 reflects growth in the Nigerian population [Table 1] and an inclusion of additional sets of survey questions and geographic areas within geopolitical regions as previously reported [11].

**Table 1:** Characteristics of the Nigeria Demographic and Health Surveys

Title of Survey	Conducted by	Coverage	Sampling frame & population size	Sampling method	Sample size (*n*)
Nigeria Demographic and Health Survey 1999	National Population Commission Abuja, Nigeria and ORC Macro Calverton, Marylands, USA	National (36 states and a federal capital territory)	1999 census with a population of 88,992,220	Two-stage stratified cluster design	8,199
Nigeria Demographic and Health Survey 2003	National Population Commission Abuja, Nigeria and ORC Macro Calverton, Marylands, USA	National (36 states and a federal capital territory)	1991 census with a population of 88,992,220	Two-stage stratified cluster design	7,620
Nigeria Demographic and Health Survey 2008	National Population Commission Abuja, Nigeria and ORC Macro Calverton, Marylands, USA	National (36 states and a federal capital territory)	2006 census with a population of 140,431,790	Two-stage stratified cluster design	33,385
Nigeria Demographic and Health Survey 2013	National Population Commission Abuja, Nigeria and ICF International, Rockville, USA	National (36 states and a federal capital territory)	2006 census with a population of 140,431,790	Three-stage stratified cluster design	38,948

### Definitions of infant and young child feeding indicators

#### Core indicators

- Early (timely) initiation of breastfeeding: The proportion of children 0–23 months of age who were put to the breast within one hour of birth.
- Exclusive breastfeeding: The proportion of infants 0–5 months of age who received breast milk as the only source of nourishment (but allow oral rehydration solution, drops or syrups of vitamins and medicines).
- Continued breastfeeding at one year: The proportion of children 12–15 months of age who were fed breast milk.
- Introduction of solid, semi-solid or soft foods: The proportion of infants 6–8 months of age who received solid, semi-solid or soft foods.
- Minimum dietary diversity: The proportion of children 6–23 months of age who received foods from four or more food groups. The seven food groups used for tabulation of this indicator are:
  - Grains, roots and tubers
  - Legumes and nuts
  - Dairy products (milk, yogurt, cheese)
  - Flesh foods (meat, fish, poultry and liver/organ meats)
  - Eggs
  - Vitamin-A rich fruits and vegetables
  - Other fruits and vegetables.
- Minimum meal frequency: The proportion of breastfed and non-breastfed children 6–23 months of age, who received solid, semi-solid or soft food (including milk feeds for non-breastfed children) the minimum number of times or more i.e. 2 times for breastfed infants 6–8 months, 3 times for breastfed children 9–23 months and 4 times for non-breastfed children 6–23 months in the previous day. “Meals” include both meals and snacks other than trivial amounts.
- Minimum acceptable diet: The proportion of children 6–23 months of age who received both minimum dietary diversity and minimum meal frequency.

#### Optional indicators

- Predominant breastfeeding: The proportion of infants 0–5 months of age who received breast milk as the predominant source of nourishment, but which allows water and water-based drinks fruit juice, ritual fluids, oral rehydration solution, syrups or drops of vitamins, during the previous day.
- Bottle-feeding rate: The proportion of infants 0–23 months of age who received any liquid including breast milk or semi-solid or soft food from a bottle with nipple/teat.
- Children ever breastfed: The proportion of children born in the last 24 months who were ever breastfed.
- Continued breastfeeding at two years: The proportion of children 20–23 months of age who were fed breast milk.
- Median duration of breastfeeding: The age in months when 50% of children 0–35 months did not receive breast milk during the previous day.

### Statistical analysis

Prevalence estimates, and corresponding confidence intervals were calculated, using sampling weights to account for the cluster sampling design employed in the NDHS [12, 14–16]. The analyses were restricted to the youngest living children aged 0–23 months who were living with their mothers aged 15–49 years [12], consistent with previously published studies [17, 18]. Based on the age criteria used to estimate each IYCF indicator and missing data, the number of mothers included in the analysis varied [Additional file 1: Table S1]. Differences in prevalence estimates in IYCF indicators were expressed as percentages comparing each survey across the study period using chi-squared to test for significant differences at *p* < 0.05. In the analysis, time trends in complementary feeding indicators were considered for the period (2003–2013) based on appropriateness of available data, consistent with a previous study [19]. For analysis relating to complementary feeding indicators, the NDHS 1999 was not included because the definitions used for these indicators such as introduction of solid, semi-solid or soft foods, minimum dietary diversity, minimum meal frequency and minimum acceptable diet, were different from those employed in NDHS 2003, 2008 and 2013. All analyses were carried out using the statistical software package, Stata version 13 · 0 (Stata Inc., College Station, TX, USA), with prevalence estimate calculated using the ‘svy’ function to allow for cluster sampling.

### Ethical considerations

The DHS project obtained the required ethical approvals from the National Health Research Ethic Committee (NHREC) in Nigeria before the surveys were conducted (Assigned Number NHREC/01/01/2007). Participants were informed of the rationale for the surveys, the time frame for the interview, confidentiality of their responses, and that, they don’t have to answer the questions if they do not feel comfortable doing so. All participants provided written informed consent before they participated in the surveys. The data used in this study were anonymous and publicly available to apply for online. Approval was sought from MEASURE DHS/ICF International and permission was granted for this use.

## Results

### Core indicators

**Fig. 1:**
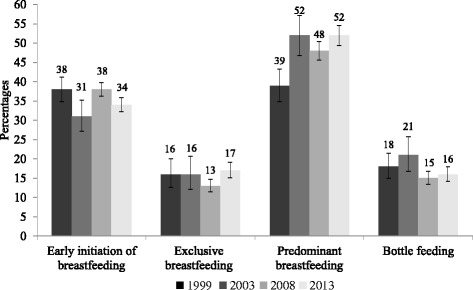
Trends in key breastfeeding practices in Nigeria, 1999–2013. Early or timely initiation of breastfeeding: The proportion of children 0–23 months of age who were put to the breast within one hour of birth. Exclusive Breastfeeding: The proportion of infants 0–5 months of age who received breast milk as the only source of nourishment (but allows oral rehydration solution, drops or syrups of vitamins and medicines). Predominant breastfeeding: The proportion of infants 0–5 months of age who received breast milk as the predominant source of nourishment (but which allows water and water-based drinks fruit juice, ritual fluids, oral rehydration solution, syrups or drops of vitamins). Bottle feeding: The proportion of infants 0–23 months of age who received any liquid (including breast milk) or semi-solid/soft food from a bottle with nipple/teat

The proportion of infants who were put to the breast within the first hour of birth ranged from 38% in 1999 to 34% in 2013 [Fig. 1]. The analysis showed that early initiation of breastfeeding decreased significantly from 1999 to 2003 by 7.0% (95% CI: −12.0, −0.2; *p* = 0.010) and 4.3% (95% CI: −8.1, −0.5; *p* = 0.028) over the study period [Table 2]. Exclusive breastfeeding and continued breastfeeding at one year remained relatively unchanged over time at 1.6% (95% CI: −2.7, 5.9; *p* = 0.478) and −0.1% (95% CI: −4.8, −2.9; *p* = 0.615), respectively) [Table 2]. The introduction of solid, semi-solid and soft food increased significantly by 8.4% (from 2003 to 2008; 95% CI: 1.2, 15.6; *p* = 0.023), a period when the IYCF policy was introduced in Nigeria. Similar results over the period (2003–2008) were evident for minimum dietary diversity which increased by 3.8% (95% CI: 0.4, 7.2; *p* = 0.030); minimum meal frequency increased by 8.7% (95% CI: 4.7, 12.6; *p* < 0.001) and minimum acceptable diet increased by 2.4% (95% CI: 0.1, 4.7; *p* = 0.037). However, minimum dietary diversity decreased significantly by 9.7% (95% CI: −13.1; −6.3; *p* < 0.001) and minimum acceptable diet decreased by 3.5% (95% CI: −5.7, −1.3; *p* < 0.002) over the period spanning (2003–2013). Minimum meal frequency increased significantly by 13.8% (95% CI: 9.9, 17.8; *p* < 0 .001) over the same period [Table 2].

**Table 2:** Prevalence differences as change in percentage-points of IYCF indicators in Nigeria (NDHS, 1999 – 2013)

IYCF indicators	2003-1999		2008- 2003		2008- 1999		2013-2008		2013-2003		2013-1999	
	% (95% CI)	*p*	% (95% CI)	*p*	% (95% CI)	*p*	% (95% CI)	*p*	% (95% CI)	*p*	% (95% CI)	*p*
Core												
Early initiation of breastfeeding	−7.0 (-12.0, -0.2)	0.010	6.7 (2.1, 11.2)	0.004	−0.4 (-4.2, -3.4)	0.829	−3.8 (-6.4, -1.3)	0.004	2.8 (-1.7, -7.3)	0.221	−4.3 (-8.1, -0.5)	0.028
Exclusive breastfeeding	0.05 (-5.4, -6.4)	0.866	−2.9 (-7.6, -1.8)	0.223	−2.4 (-6.6, -1.7)	0.256	4.0 (1.2, 6.7)	0.004	1.0 (-3.9, 5.9)	0.676	1.6 (-2.7, 5.9)	0.478
Continued breastfeeding at 1 year	5.0 (-0.1, -0.2)	0.057	−4.5 (-8.9, -0.1)	0.044	0.5 (-3.2, -4.3)	0.780	−1.5 (-4.3, -1.3)	0.290	−6.0 (-0.4, -1.6)	0.008	−0.1 (-4.8, -2.9)	0.615
Introduction of solid, semi-solid and soft food	N/A	N/A	8.4 (1.2, 15.6)	0.023	N/A	N/A	−4.2 (-8.4, 0.01)	0.051	4.2 (-3.0, 11.4)	0.257	N/A	N/A
Minimum dietary diversity	N/A	N/A	3.8 (0.4, 7.2)	0.030	N/A	N/A	−13.5 (-15.6, -11.3)	<0.001	−9.7 (-13.1, -6.3)	< 0.001	N/A	N/A
Minimum meal frequency	N/A	N/A	8.7 (4.7, 12.6)	< 0.001	N/A	N/A	5.2 (2.8, 7.5)	< 0.001	13.8 (9.9; 17.8)	< 0.001	N/A	N/A
Minimum acceptable diet	N/A	N/A	2.4 (0.1, 4.7)	0.037	N/A	N/A	−5.9 (-7.2, -4.5)	< 0.001	−3.5 (-5.7, -1.3)	0.002	N/A	N/A
Optional												
Children ever breastfed	2.1 (0.4, 3.8)	0.013	−0.5 (-1.9, -0.8)	0.415	1.6 (0.3, 2.8)	0.013	−7.9 (-9.2, -6.7)	< 0.001	−18.5 (-20, -16.8)	< 0.001	−16.4 (-18.0, -14.8)	<0.001
Continued breastfeeding at 2 years	−2.1 (-11.6, 7.5)	0.673	−1.2 (-9.2, 6.7)	0.758	−3.3 (-10.7, 4.1)	0.383	2.8 (-2.3, 7.8)	0.284	1.5 (-6.3, 9.4)	0.705	−0.5 (-7.9, -6.8)	0.888
Predominant breastfeeding	12.9 (6.0, 19.7)	< 0.001	−3.4 (-9.5, 2.7)	0.277	9.4 (4.3, 14.5)	< 0.001	3.7 (-0.1, 7.4)	0.056	0.3 (-5.9, 6.4)	0.934	13.1 (7.9, 18.3)	<0.001
Bottle-feeding	2.4 (-0.3, 5.2)	0.086	−3.5 (-5.7, -0.1)	0.006	−0.9 (-2.8, 0.1)	0.338	1.5 (0.1, 2.9)	0.040	−1.9 (-4.3, 0.6)	0.133	0.6 (-1.4, 2.6)	0.576

%: Percentage change

95% CI: 95% lower and upper levels of Confidence Interval

N/A: Data not appropriate for analysis based on variation in definition of the indicators in the 1999 NDHS

IYCF: Infant and young child feeding practices

### Optional indicators

A significant decrease in children ever breastfed was observed during the study period, a decrease of 16.4% (95% CI: −18.0; −14.8; *p* <0.001). Predominant breastfeeding increased significantly by 13.1% (95%CI: 7.9; 18.3; *p* < 0.001) from 1999 to 2013, while continued breastfeeding at two years and bottle feeding remained relatively unchanged [Table 2]. The median duration of breastfeeding of all the children born in the five years preceding the surveys, was 18 months in 2003, 17 months in 2008 and 17 months in 2013, as reported in the Nigeria Demographic and Health Surveys reports compared to more than 24 months recommended.

## Discussion

### The impact of policies on the findings

In the present study, early initiation of breastfeeding (EIBF) decreased significantly in the period following the introduction of the BFHI and the National Breastfeeding policy (in 1992 and 1998, respectively). EIBF also worsened significantly over the study period, while exclusive breastfeeding (EBF) remained low in Nigeria. Predominant breastfeeding, a risk factor for diarrhoeal-related morbidity and mortality in Nigeria [12], increased over time. Children ever breastfed decreased significantly over time, which may reflect the reduction in EIBF. The study also found that complementary feeding patterns in Nigeria were low, except for minimum meal frequency, where a significant increasing trend was evident. However, a frequent feeding rate based on a 24 h dietary recall does not reflect the appropriate timing of the introduction of solid, semi-solid and soft food, diversity of food, energy density and quality of the diet [19]. Previous studies from other developing countries such as Bangladesh and Sri Lanka, reported higher complementary feeding prevalence compared to Nigeria [20]. Similarly, higher prevalence in complementary feeding practices has been reported in both Anglophone (Liberia and Ghana) and Francophone (Senegal and Niger) West African countries compared to Nigeria [21]. Various reasons have been suggested for these differences including, cultural differences among community members, infants are restricted from eating certain foods, a lack of financial resources [21] and low maternal education [20].

Globally, when compared to other developing countries such as Ghana [22] and Indonesia [23], early initiation of breastfeeding and exclusive breastfeeding rates among children aged 0–23 months and 0–5 months respectively, were lower in Nigeria. In contrast, the EIBF and EBF rates in Nigeria were higher compared to South Africa, with a similar emerging market, a growing middle class and significant resource based economy [24]. The low breastfeeding figures reported in South Africa have been attributed largely to the prenatal intent of mothers not to breastfeed because of fear of maternal to child transmission of HIV [25]. Although the proportion of mothers who bottle fed their children remained unchanged over time, an increasing trend in predominant breastfeeding was observed. In Nigeria, it is important to note that mothers from higher socio-economic status (SES) groups are more likely to bottle feed compared to mothers from lower SES group because they are more likely to be employed, have more access to advertising materials and have the financial resources to purchase breast milk substitutes [11]. The violation of the International Code of Marketing of Breastmilk Substitutes (the Code) has been reported in many countries, including Nigeria [26, 27]. Major reasons given why mothers from higher SES group bottle feed their babies include poor policy implementation of the Code and difficult work environments that do not support breastfeeding mothers [11, 28]. Previous studies from Southeast Asia were consistent with this finding, where bottle feeding was associated with higher socio-economic development of mothers [23, 29].

The WHO/UNICEF introduced the BFHI to promote and support to breastfeeding mothers at health facilities. However, a large number of women in many developing countries deliver their babies at home [30], which provides a serious challenge for the full achievement of the BFHI. In Nigeria, many women (~64%) deliver their babies at home, assisted either by a traditional birth attendant or a relative, and often are not trained in providing appropriate infant feeding options to new mothers [10, 12]. This mismatch between policy recommendation from international organisations and context specific evidence could be a reason for the low IYCF practices observed in Nigeria. Additionally, many health care professionals in Nigeria are not adequately trained in providing optimal breastfeeding options to mothers as recently reported [28]. Recent assessment of the BFHI status in Nigeria found that only a small proportion of hospitals, 8% as reported by UNICEF [9] and 95 out of 25,000 hospitals, or 0.004%, as reported by Nigerian Ministry of Health [28] were BFHI certified. This important aspect of the BFHI has practically “failed”. Nigeria has well developed IYCF legislation, health system level actions, and community level strategies and communication, including appropriate monitoring and evaluation structures to improve IYCF practices [9]. However, poor political will and non-functional or nonexistent subnational government committees to fully implement key IYCF policies, such as the BFHI, National Breastfeeding Policy, National IYCF policy among others, are likely additional reasons for Nigeria’s low IYCF practices [28].

In low and middle income countries, including Nigeria, government health expenditure as a source (GHE-S) can underpin public health policies and intervention strategies [31]. However, the Federal Government of Nigeria spends less than 15% of the annual budget on health which is below the commitment made by African Union leaders, including Nigeria, to allocate at least 15% of their annual budget to improve the health sector. Given this funding deficiency in Nigeria’s health care system, it was estimated that Nigeria would require approximately USD600 million annually to improve child health programs [32]. Even though Nigeria’s National Legislation and Health System Framework to promote IYFC practices has been described as “appropriate”, GHE-S in Nigeria is much lower compared to many sub-Saharan African countries such as South Africa, Ghana, Niger and Kenya [33]. Based on this deficit, Developmental Assistance for Health (DAH) from international donors has been a stimulus for a range of public health programs and remains a major source of funding for health initiatives in many developing countries, such as Nigeria [31]. For example, in 2012, the Federal Government of Nigeria launched the 'Saving One Million Lives' by 2015 Initiative [34], but the funding for this project was not available until 2015 when the World Bank, a major donor of DAH for Nigeria announced USD500 million to fund the initiative [35]. The mismatch in timing between the Nigerian initiative and the commencement of World Bank funding reflects gaps in health intervention funding in Nigeria, an aspect of public health intervention financing that could be extrapolated to other developing countries that receive DAH.

**Fig. 2:**
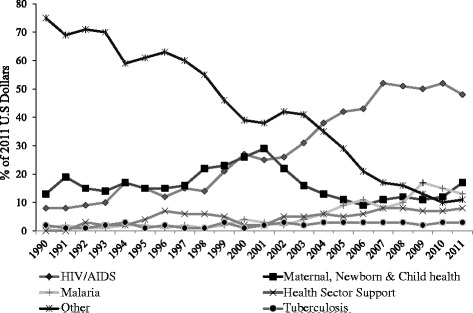
Change in the proportion of health focus areas of development assistance for health in Sub-Saharan Africa, 1990–2011. Source: IHME DAH Database 2013. DAH for other health focus areas not yet tracked by IHME was coded as “other”

In recent years, however, there has been a shift in DAH, quite appropriately given the potential disease burden associated with HIV, from maternal, newborn and child health interventions to HIV initiatives [Fig. 2]. This shift was in response to donor agency’s perceived health priorities in Sub-Saharan Africa, particularly in countries (including Nigeria), where HIV is endemic [36]. Although, the focus on HIV initiatives has been beneficial, this has mainly drawn resources away from initiatives that target child health interventions (including nutrition) in these countries. This has been the case for countries such as Nigeria, Africa’s most populous country and largest economy, with 40 million children [37], and the largest recipient of DAH in sub-Saharan Africa [36]. These funding gaps could be reasons for why optimal IYCF practices in Nigeria are lagging behind IYCF policies recommended by the Government of Nigeria.

Furthermore, based on analyses of nationally representative data, additional reasons why IYCF practices have remained low include socio-economic factors such as low maternal education and poor household wealth, health service factors such as fewer antenatal care visits and home delivery and individual factors such as younger maternal and child age. A more detailed discussion on the impact of these factors on suboptimal IYCF practices in Nigeria has been described previously [10, 11, 19, 38]. Evidence from regional Nigeria also found that a lack of family support, mother’s employment, and myths and belief systems held for breastfeeding contribute to suboptimal IYCF practices in Nigeria [39, 40]. A lack of transparency and poor accountability of GHE–S and DAH expenditure in Nigeria have also been flagged as reasons for why IYCF policies have basically “failed” in improving IYCF practices in Nigeria [41, 42].

### Going forward

The current initiatives by the Federal Government of Nigeria, such as the Subsidy Reinvestment and Empowerment Programme Maternal and Child Health Program (SURE-P MCH) [43, 44] and “Saving One Million Lives Initiative” (SOML) [35] are interventions that are needed on a large scale to improve childhood feeding practices in Nigeria. Evidence from this study indicates that a revision and assessment of policy responses to date, and the full implementation of context specific and cost effective interventions are necessary to improve infant and young child nutrition in Nigeria. Improvements in the IYCF practices would result in the achievement of the Global Nutrition Target by 2025, where Global Target 5 is concerned with increasing the rate of exclusive breastfeeding in the first six months up to at least 50% by the year 2025 [45]. Scaling up efforts to achieving these targets will require initiatives at the health system and community levels, and combinations of integrated and stand-alone policies that are well implemented, and galvanised by broader political commitment at all levels in Nigeria [46]. Availability of appropriate weaning and toddler foods may be a factor in the decline of minimum dietary diversity and minimum acceptable diet. The circumstances of food availability merit investigation to inform government policy and initiatives. Achieving the above mentioned targets would not only improve child nutrition and health, but are also likely to have wider economic gains, associated with reduced healthcare costs and improved worker productivity due to a decrease in child sick days [45].

The implications of the impact, or otherwise, of national policies on trends in IYCF practices is that the Nigerian government needs to provide adequate funding for the health care sector, consistent with the resolution of the Abuja Declaration, and streamlined with current fiscal allocations. Health initiatives must be designed to target the local community, and health care spending in the Nigerian context must be transparent, monitored and evaluated periodically given the mismanagement of previous funds [42]. Furthermore, the work environments in which women work, particularly the growing middle class, and the influence of grandmothers, both maternal or paternal, have also been reported as providing major obstacles to optimal infant feeding practices in Nigeria [39, 47]. Workplace changes in the form of legislation, such as the extension of the paid maternity leave from three to six months for female workers and paid paternity leave, was recently announced by a regional Nigerian government and are needed to improve IYCF practices in Nigeria. Economic incentives to employers to provide environments conducive to breastfeeding, such as provision of crèches or breastfeeding breaks are also required nationally and at all level of government, including the private sector, to improve breastfeeding patterns of Nigerian mothers.

In many Nigerian communities, family members particularly grandmothers and husbands, have a major influence on new mothers and provide support to mothers post-delivery, but their advice may reflect cultural beliefs that do not usually promote optimal infant feeding practices [11, 48]. Community based initiatives which have been successful in regional Nigeria and Ghana [49, 50] that involve family members in health information sessions, and tailored to the specific socio-cultural context in which women live, would maximise positive results in Nigeria. In addition, facility based determinants have also been reported as major impediments for appropriate feeding patterns in Nigeria [11, 19]. Globally, various reports have shown that the BFHI has been influential in promoting optimal infant and child feeding practices in many communities [51–56]. Strengthening the BFHI with the establishment of subnational IYCF committees with appropriate funding in Nigeria, and training of health professionals, including the incorporation of the baby friendly community initiative are also interventions needed to improve feeding practices of Nigerian mothers. The community and facility based determinants for suboptimal feeding practices identified in Nigeria have also been flagged in other developing countries [17, 57, 58]. Thus, initiative and policy implementation plans from these findings could have wider relevance to other emerging countries with low IYCF indices.

Sustainable Development Goals (SDG) 2 and 3, advocate for improved nutrition, and healthy lives for all [57]. Good health starts with higher uptake of breastfeeding and nutritionally adequate foods at weaning. To fulfil these and other SDGs, Nigeria could design and implement context specific initiatives and policies that adequately supports a healthcare system, promotes optimal infant and child feeding practices, appropriately regulates marketing of baby foods, and produces mother-friendly workplace legislation that considers the specific physical and socio-cultural contexts in which mothers raise their children.

The following limitations should be considered when interpreting the study findings. Firstly, information on IYCF practices was based on self-reporting, where mothers recall how the child was fed during the periods referred to by the survey questions. Secondly, the study was based on a description of IYCF indicators at the national level. Nevertheless, it is important to have estimates for each indicator at the community and household level with regard to socio-economic characteristics to guide context specific interventions as reported in previous studies [11, 19]. Thirdly, there is no sufficient data on the characteristics of the BFHI and other national and subnational policies to adequately specify the scope and duration of the implementation of these policy responses, nor is there contemporaneous baseline data or pre-policy data in order to quantitatively correlate the implementation of a policy change in IYCF practices. The 1999 NDHS was conducted almost 10 years after the first major national policy intervention (BFHI) was launched in Nigeria to improve breastfeeding practices during a period when Nigeria was under authoritarian regimes with minimal attention given to the health care sector [59]. While there is not a formal correlation between policy implementation and IYFC trends, this paper provides a detailed exposition of how likely the IYCF practices have changed in the epoch post national BFHI in the context of the policy implementation in Nigeria as well as suggests attempts for improvement based on previous reports. Other key strengths of the study were that the samples were nationally representative. Also that the study used important data source on recommended infant and young feeding indicators and the statistical adjustment made in the analysis, the restriction of the analysis to the youngest living children aged 0–23 months living with their mothers aged 15–49 years to reduce recall bias as conducted previously [18], and the high response rates.

## Conclusion

Over a decade, the Federal Government of Nigeria introduced various policies to promote and support infant and child feeding (IYCF) practices. However, these broader policy strategies are not reflected in the outcome as measured by nationally representative prevalence estimates of IYCF practices, where key breastfeeding and complementary feeding practices remain below expected levels.

A comprehensive strategic plan that strengthens the involvement of the community and health care professionals, an improvement in the political resolve at all levels, as well as more accountability in health care expenditure is proposed as an adjunct to current interventions and policies to improve infant and young child feeding practices in Nigeria.

## Additional file

Additional file 1: Table S1. Numbers of mothers included in analyses based on the definitions of IYCF indicators by year (NDHS, 1999-2013). (DOCX 17 kb)

## Abbreviations

DAH: Development assistance for health
IHME: Institute for health metric and evaluation
IYCF: Infant and child feeding
UNICEF: United Nation Children’s Fund
WHO: World Health Organisation
