# Supplementary material for: Have policy responses in Nigeria resulted in improvements in infant and young child feeding practices in Nigeria?
Source: Int Breastfeed J. 2017 Feb 8;12:9. doi: 10.1186/s13006-017-0101-5 (PMC5299643; doi:10.1186/s13006-017-0101-5)
Supplement: Additional file 1: Table S1. — Numbers of mothers included in analyses based on the definitions of IYCF indicators by year (NDHS, 1999-2013). (DOCX 17 kb) [file 13006_2017_101_MOESM1_ESM.docx]

**Additional file 1: Numbers of mothers included in analyses based on the definitions of IYCF indicators by year (NDHS, 1999-2013)**

|  | **1999 (N = 8,199)** | | **2003 (N = 7,620)** | | **2008 (N = 33,385)** | | **2013 (N = 38,948)** | |
| --- | --- | --- | --- | --- | --- | --- | --- | --- |
| IYCF Indicators | **n*** | **% [95% CI]** | **n*** | **% [95% CI]** | **n*** | **% [95% CI]** | **n*** | **% [95% CI]** |
| **Core** |  |  |  |  |  |  |  |  |
| Early initiation of breastfeeding (0-23 months) | 2167 | 38.1 (35.0-41.4) | 2248 | 31.0 (27.2-35.2) | 10225 | 37.7 (36.0- 39.4) | 11712 | 33.9 (32.1-35.7) |
| Exclusive breastfeeding (0-5 months) | 557 | 15.9 (12.5-20.0) | 658 | 16.4 (12.6-21.1) | 2832 | 13.5 (11.9-15.2) | 2926 | 17.4 (15.5-19.5) |
| Continued breastfeeding at 1 year (12-15 months) | 570 | 85.1 (81.8-87.9) | 386 | 90.1 (85.7-93.3) | 1988 | 85.66 (83.8-87.4) | 2373 | 84.1 (82.1-86.0) |
| Introduction of solid, semi-solid and softs (6-8 months) | N/A | N/A | 352 | 60.5 (54.0-66.6) | 1505 | 68.8 (66.0-71.6) | 1653 | 64.6 (61.6-67.6) |
| Minimum dietary diversity (6-23 months) | N/A | N/A | 1590 | 26.1 (23.2-29.3) | 7393 | 29.9 (28.4-31.5) | 8786 | 16.4 (15.1-17.9) |
| Minimum meal frequency (6-23 months) | N/A | N/A | 1590 | 41.5 (38.0-45.0) | 7393 | 50.1 (48.6-51.9) | 8786 | 55.3 (53.6-57.0) |
| Minimum acceptable diet (6-23 months) | N/A | N/A | 1590 | 10.8 (8.9-13.0) | 7393 | 13.2 (12.2-14.2) | 8786 | 7.3 (6.5-8.3) |
| **Optional** |  |  |  |  |  |  |  |  |
| Children ever breastfed (0-24 months) | 2245 | 93.3 (92.1-94.4) | 2343 | 95.4 (94.0-96.5) | 10621 | 94.9 (94.4-95.3) | 12181 | 76.9 (75.8-78.0) |
| Continued breastfeeding at 2 years (20-23 months) | 236 | 36.0 (30.3-42.2) | 247 | 34.0 (27.7-40.9) | 1085 | 32.7 (29.5-36.2) | 1456 | 35.5 (32.3-38.9) |
| Predominant breastfeeding (0-5 months) | 557 | 38.6 (34.5-43.0) | 658 | 51.5 (46.3-56.7) | 2831 | 48.1 (45.7-50.5) | 2926 | 51.8 (49.1-54.4) |
| Bottle-feeding (0-23months) | 2167 | 12.1 (10.6-13.8) | 2248 | 14.5 (12.5-16.8) | 10225 | 11.2 (10.3-12.1) | 11712 | 12.7 (11.7-13.7) |

N = sample size

n*: number of cases of IYCF indicator by year

%: proportions of mothers who practiced each IYCF indicator

95% CI: 95% lower and upper levels of Confidence Interval (CI)

N/A: data not appropriate for analysis based on variation in definition of the indicators in the 1999 NDHS
